# Remediation of Punching Shear Failure Using Glass Fiber Reinforced Polymer (GFRP) Rods

**DOI:** 10.3390/polym13142369

**Published:** 2021-07-19

**Authors:** Ekkachai Yooprasertchai, Ratchanon Dithaem, Titi Arnamwong, Raktipong Sahamitmongkol, Jira Jadekittichoke, Panuwat Joyklad, Qudeer Hussain

**Affiliations:** 1 Construction Innovations and Future Infrastructure Research Center (CIFIR), Department of Civil Engineering, Faculty of Engineering, King Mongkut’s University of Technology Thonburi, Bangkok 10140, Thailand; ekkachai.yoo@kmutt.ac.th (E.Y.); Dithaemnin@hotmail.com (R.D.); pathong4646@hotmail.com (T.A.); raktipong.sah@kmutt.ac.th (R.S.); jira_pj123@hotmail.com (J.J.); 2 Department of Civil and Environmental Engineering, Srinakharinwirot University, Nakhon Nayok 26120, Thailand; panuwatj@g.swu.ac.th; 3 Center of Excellence in Earthquake Engineering and Vibration, Department of Civil Engineering, Chulalongkorn University, Bangkok 10330, Thailand

**Keywords:** flat slab, punching shear failure, GFRP rods, ductility, energy dissipation capacity

## Abstract

The results of an experimental program on shear-strengthening of flat slabs using Glass Fiber Reinforced Polymer (GFRP) rods are presented. A total of seven specimens were tested under an upward concentric monotonic loading until failure. One specimen served as a control and was tested without any modification. The remaining six specimens were strengthened with post-installed GFRP rods in single (SG), double (DB), and radial (RD) patterns within shear critical parameters around the centric column. The results of this experimental study suggest that GFRP rods are capable of enhancing both the peak load and deformation capacity. Furthermore, brittle failure associated with punching shear failure was successfully avoided by all strengthening patterns. Of all of the patterns, the RD pattern resulted in maximum peak load increase and corresponding deformation capacity while the lowest bound was created by the SG pattern. The results suggested that SG, DB and RD patterns enhanced ultimate loads up to 9.1, 11.3 and 15.7% while corresponding deflections increased up to 109, 136 and 154%. Strain measurement on flexural reinforcement suggested that all strengthened specimens were able to withstand higher longitudinal strains than yield. It was further shown that reducing the spacing between the GFRP rods efficiently enhanced peak loads, nevertheless, neither this change was proportional, nor did it result in an enhanced energy dissipation capacity. In the end, recommendations of American Concrete Institute (ACI) for the shear strength of two-way systems were modified to incorporate the contributions from GFRP rods. The results indicate that the proposed analytical approach provides an excellent match with the experimental results.

## 1. Introduction

Flat slab is a two-way load distributary system that directly rests on columns and its use has been found more recurrent in recent times [1], mainly attributed to large flooring heights and the reduced construction times associated with it. The direct load transfer mechanism renders the supporting columns to act as upward concentrated loads. This effect is detrimental in the sense that it challenges the inherent shear capacity of the slab. The latter often comes second resulting in inclined cracks originating at the slab column’s intersecting surface. Nowadays, a significant number of flat slabs need to be strengthened [2]. This demand may arise from several factors that include construction or design errors, disobeying code provisions, environmental deterioration of materials, or increase in gravitational load [3]. Flat slabs are vulnerable to punching shear failure and many such circumstances have been reported [4,5,6]. This insufficient shear capacity of concrete puts great demands on the use of additional shear reinforcement to counter punching shear failure and prevent the loss of structural integrity.

Several methods to strengthen inherently insufficient flat slabs include post-installation of shear reinforcement, enlarging the column periphery near the slab either by drop panel or column capitals [7,8,9,10]. Both the column capitals and drop panels result in a larger support area for flat slabs than that provided by bare columns. The bifold outcomes are the reductions both in shear stress and the required slab thickness. However, the aesthetics are compromised as it requires access to the upper slab face which is usually covered by the floor. Post-installation of shear reinforcement leaves undetected marks, slight appearance changes and can be practical in many cases in comparison to either drop panels or column capitals [11]. Composite in nature, FRP was rated 8 to 10 times stronger in tension than common steel reinforcement [12]. Brittle in behavior, it exhibits a unique tensile strength higher than steel with their weight equal to one-quarter of steel [13]. Field applications have shown excellent performance and durability of FRP retrofitted structures [14,15,16,17,18,19,20,21,22,23,24,25]. FRP strips were bonded “On Grooves” (EBROG) and “In Grooves” (EBRIG). The strengthening pattern comprised of 1- or 2-layer FRP strips bonded under slabs on two grooves which were 4 or 8 mm wide and depth ranged from 8–12 mm. Results demonstrated that such FRP patterns were able to enhance punching shear strength up to 60% for EBROG while it grew up to 28% for EBR [26].

In the event when both the upper and lower slab surfaces are accessible, Glass Fiber Reinforced Polymer (GFRP) rods can be installed in holes pierced through slabs with the assistance of epoxy adhesives. The resulting system has shown promising results without compromising on the architecture of the system. In a similar manner, Carbon Fiber Reinforced Polymer (CFRP) fans can be installed through the holes drilled inside the slab and the resulting failure was effectively altered from shear to flexure [27,28,29,30]. GFRP is a common type of composite material whose fibers can be randomly arranged, flattened into a sheet, or woven into a fabric [31,32,33]. Its ductility was found to be greater than CFRP [34]. GFRP-reinforced two-way flat slabs indicated smaller post-cracking stiffness, larger crack widths, and smaller punching shear capacities than their equivalent steel reinforced slabs when identical reinforcement amounts were employed. This is ascribed to their smaller axial stiffness compared to steel bars [35]. GFRP bars also possess the advantage of being cheaper than composite materials made up of carbon or aramid [36,37,38].

Recently, GFRP was utilized in composite tube columns along with recycled aggregate concrete to furnish excellent compressive strength [39]. Despite possessing brilliant inherent attributes, the use of GFRP as a shear reinforcement is very limited. GFRP was compared to steel reinforcement as shear reinforcement, and it was concluded to have comparable improvements in punching shear strength [40]. Other studies also incorporated GFRP reinforcement as a replacement of steel reinforcement and concluded to have parallel enhancements in punching shear strength of flat slabs together with the alteration of failure mode from shear to flexure [31,32,33,34,35,36,37,38,39,40,41,42,43]. Till now, GFRP bars have shown substantial potential as a suitable alternative to conventional steel shear reinforcement in flat slabs [34,35,36,37]. However, its prospective in terms of post-installed shear enhancer is yet to be explored. The main objectives of this study are to explore the potential of GFRP as post-installed shear reinforcement, to investigate their installing patterns considering shear strength, ductility, and failure modes and to investigate the effect of shear critical section perimeter by varying the number of peripheral rings. It is to be noted that parameters such as the diameter of the GFRP rods and flat slab thickness are not varied in this study.

## 2. Experimental Program

### 2.1. Materials

**Figure 1:**
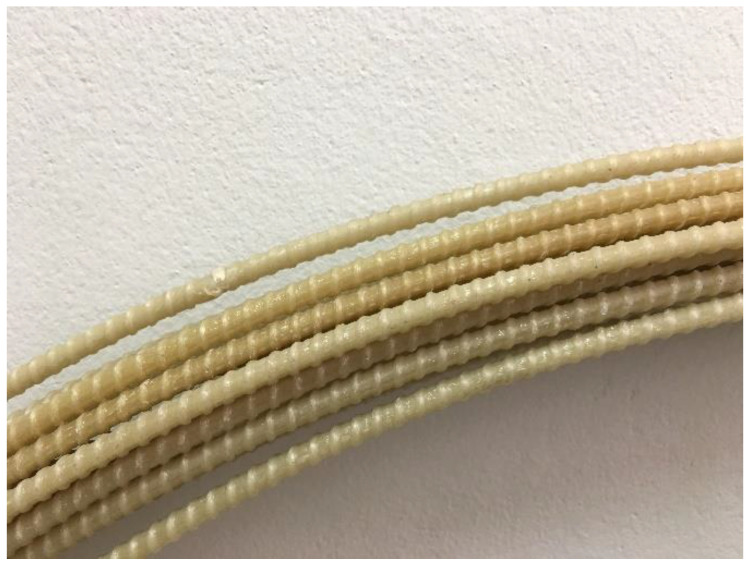
Typical GFRP rods.

Normal weight and ready mixed concrete with a mean compressive strength of 23.5 MPa was used. Concrete comprised of Type I Portland cement, the maximum aggregate size of 25 mm and slump was estimated to be 75 ± 25 mm. The tensile strength test of the GFRP rods was carried out as per American Society for Testing and Material (ASTM D7205/D7205M-06) [44]. The resin used to fill the drilled holes in the slab was High Performance 2-Part Epoxy resin produced by Smart and Bright Co., Ltd. (Bangkok, Thailand) It consisted of part A and B which were mixed in 2:1 to yield the final product. Flexural reinforcement used was 16 mm steel bars delivered by Siam Yamato Steel Co., Ltd. (Rayong, Thailand) All steel bars came from a single lot. A few steel bar specimens were randomly selected and the tensile tests were performed to yield their mechanical properties. The mechanical properties of reinforcement are specified in Table 1. Typical GFRP rods are shown in Figure 1.

### 2.2. Test Specimens

**Figure 2:**
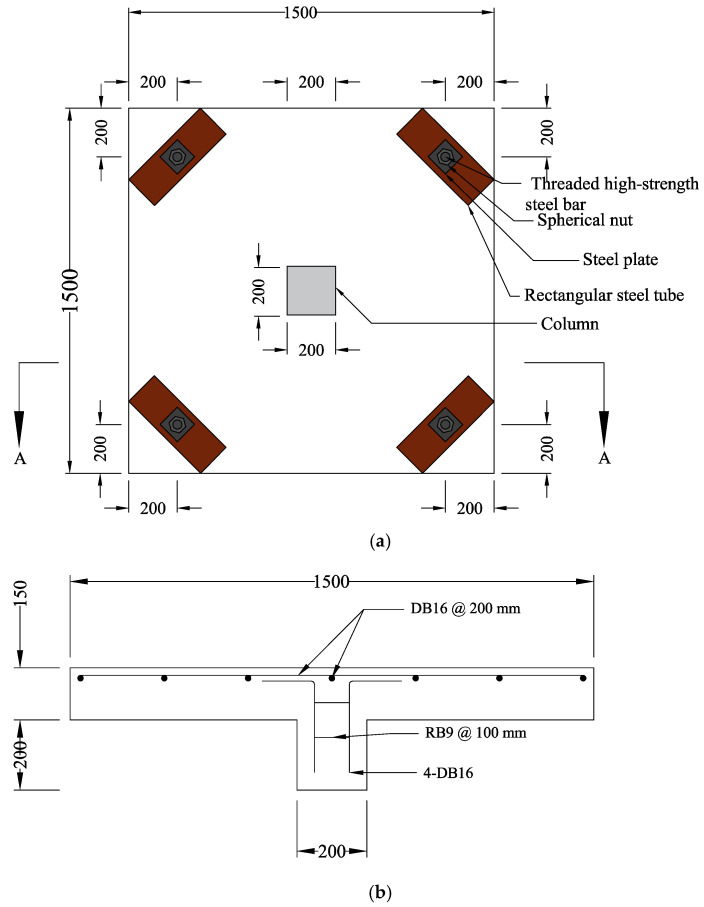
(**a**) Typical details of test specimen (units: mm); (**b**) Section AA details (units: mm).

A total of 7 flat slab specimens were tested in this study with a typical specimen designed to be as close as the real structure. To ensure the behavior and loading conditions simulating a real system, a slab-column connection shape was adopted. However, owing to the limitations of testing apparatus and handling issues, a scaled-down system was adopted. Slabs measured 1500 mm by 1500 mm in plan with a thickness of 150 mm. Column dimensions were 200 mm by 200 mm cross-section and a depth of 200 mm. The flexural reinforcement ratio of the specimen was designed and controlled to be 0.0084, which falls in the same range of actual structures, i.e., 0.0035 to 0.02. A nominal cover thickness of 20 mm was provided in the slabs. Flexural reinforcement of the slabs in orthogonal directions was provided at a center to center spacing of 200 mm. A typical layout of the slab specimen and flexural reinforcement details are shown in Figure 2.

### 2.3. Strengthening Plan

**Figure 3:**
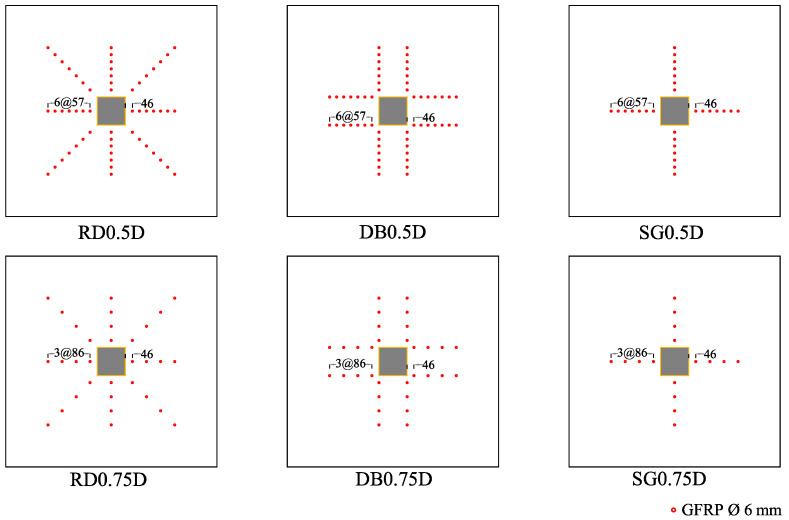
Pattern details of the GFRP rods.

Seven specimens included one control and the remaining six were classified into two groups depending upon shear reinforcement spacing. Each subgroup contained 3 specimens with different GFRP rod patterns as depicted in Figure 3. The first and second groups had a GFRP rod spacing of 0.5 and 0.75 times of slab effective depth, respectively. Specimen ID is read in order: GFRP rods pattern (SG for single pattern, DB for double pattern and RD for radial pattern) followed by their spacing (0.5D and 0.75D). For instance, SG0.75D corresponds to the specimen with a single pattern of GFRP rods at spacings of 0.75 times the slab’s effective depth. It is worth mentioning that the first GFRP rod was placed at a distance of 0.4 times the slab’s effective depth from each column’s face. Further details are presented in Table 2.

### 2.4. Specimen Preparation

**Figure 4:**
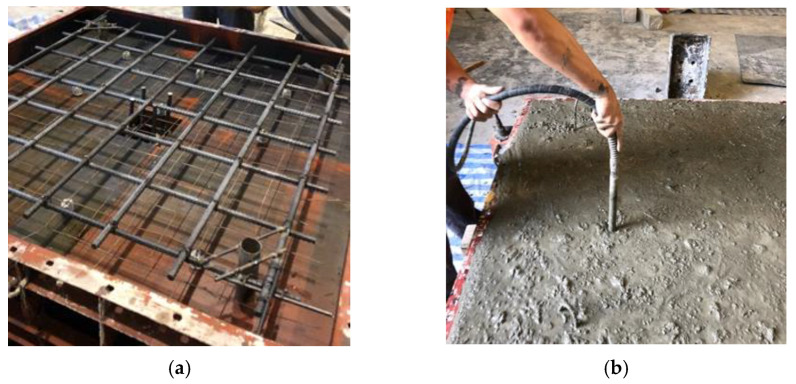
Specimen preparation (**a**) steel wire mesh (**b**) placement of concrete.

**Figure 5:**
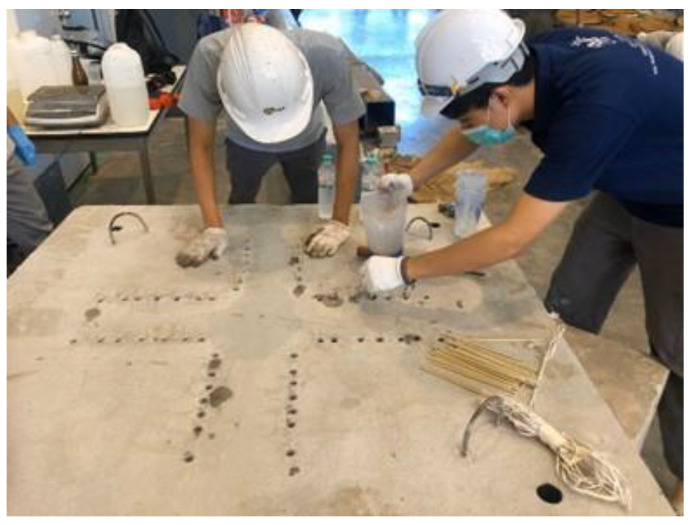
Installation of the GFRP rods.

DB-16 deformed bars were cut to a length of 1350 mm each to be used as flexural reinforcement. Steel tubes were welded to main bars to serve as chairs providing a concrete cover of 25 mm and were positioned at four corners of steel mold at an offset of 200 mm. Furthermore, steel tubes also served as holes for anchorage. After fixing the steel, 2 strain gauges each with a gauge length of 5 mm were installed along the central main bars at a distance of 0.5d from the column’s face. For this installation, the steel surface was first cleaned with acetone in one-way motion. A special glue CC-33A was deployed to fix strain gauges onto the steel bar surface. Finally, strain gauges were covered by rubber tape to preclude water intrusion. Concrete was first poured into the column section and a mechanical vibrator was utilized to attain proper compaction. Concrete bleeding was observed for 30 min after pouring and that was eventually followed by plastering surface. Curing in the first 24 h was performed by covering the surfaces using plastic sheets to prevent any moisture loss in form of evaporation. Normal curing was maintained for 28 days. Figure 4 illustrates the preparation of an individual specimen. Post-installation of the GFRP rods was accomplished by drilling holes of 14 mm through slabs at particular locations around the column’s periphery. The GFRP rods were passed through the holes before sealing the holes completely with resin (Figure 4 and Figure 5).

### 2.5. Loading and Instrumentation

**Figure 6:**
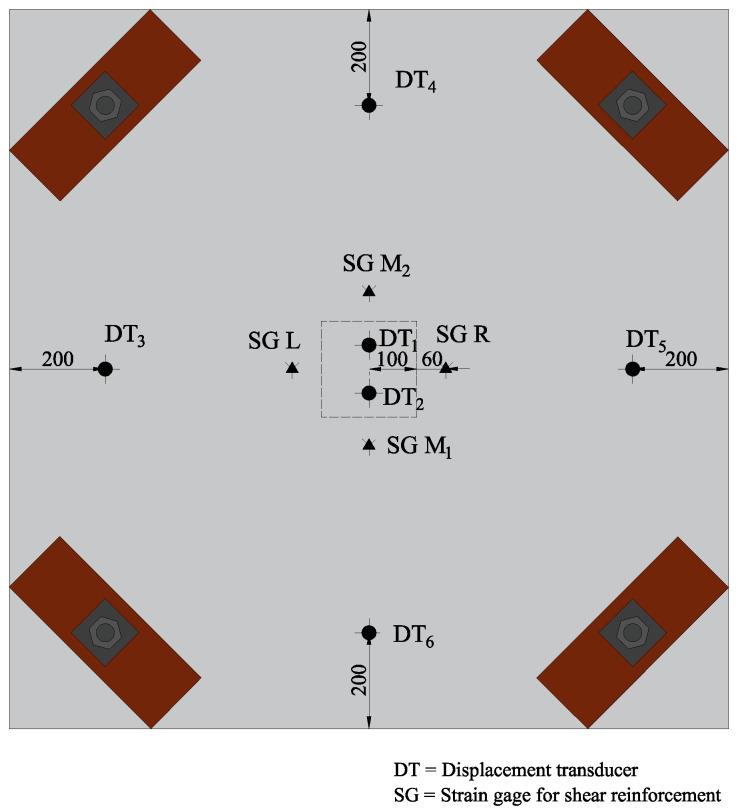
Installation of transducers and strain gauges.

Steel anchors were passed through steel tubes and on one side of the slab, they were anchored to a steel beam to act as support whilst they were bolted to a square steel section on the other side of the slab. Scaffolding was provided to be a support for two steel beams whose function was to facilitate the installation of Linear Variable Differential Transformers (LVDTs) for deflection measurement. A total of 6 LVDTs were deployed in this study. Out of 6, 2 were attached at the slab’s center where maximum deflection was anticipated. The rest were attached one at each midway between the line of supports. Figure 6 shows the locations of the LVDTs mounted on the slab’s top surface. Tips of LVDTs were not directly attached to the concrete surface. Rather, small pieces of Compact Disks (CDs) were glued onto the concrete surface to furnish a smooth surface for measurement. Four strain gauges were deployed to monitor the strain of longitudinal steel bars. Two of them were mounted on top while the rest two were mounted on bottom bars. Similarly, 4 gauges monitored strain of shear reinforcement one on each side of column (see Figure 6 marked with SGL, SGR, SGM1 and SGM2).

**Figure 7:**
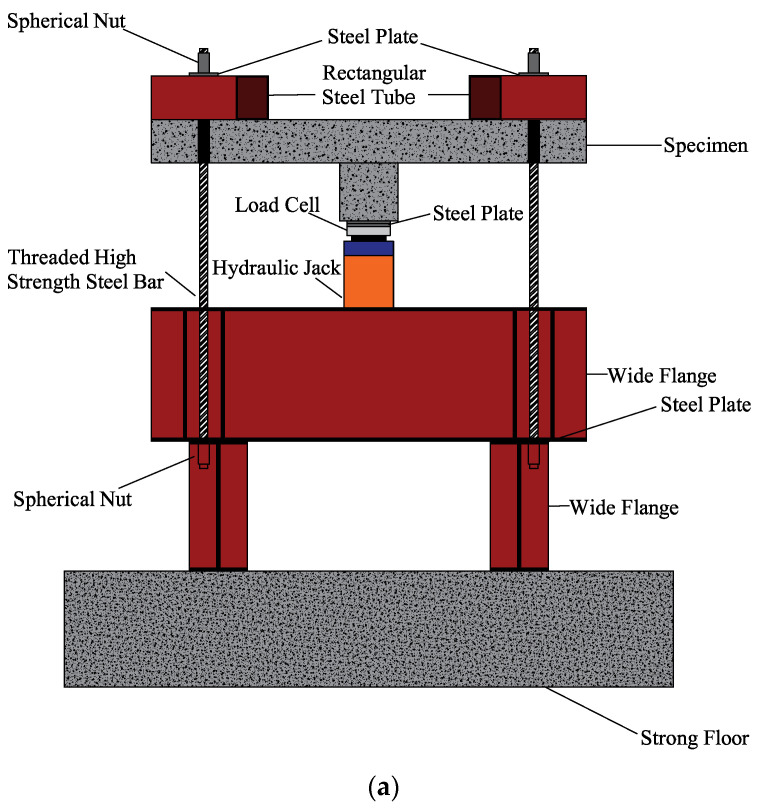

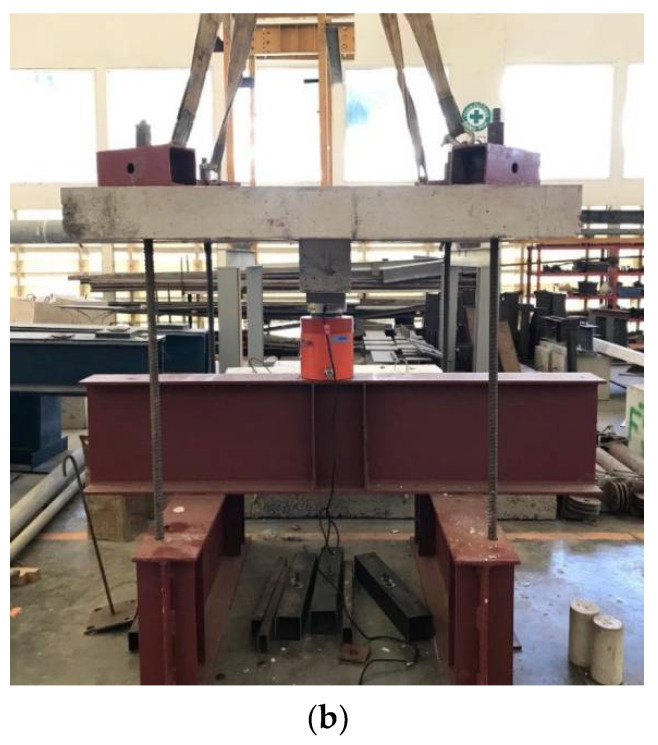
(**a**) Schematic representation of test setup; (**b**) Actual test setup.

In real structures, a column in flat slabs acts as a concentrated load that eventually sparks punching shear failure. This was impersonated by loading the column below with the load cell in combination with a hydraulic jack of 500 kN capacity. The load cell was placed on the supporting beam on top of which rested the loading plate. Figure 7a describes the schematic view of loading assembly, and the actual test setup is demonstrated in Figure 7b. A displacement control load was applied to each specimen at a rate of 1 mm/min until failure.

## 3. Test Results

### 3.1. Load Capacity and Failure Modes

**Figure 8:**
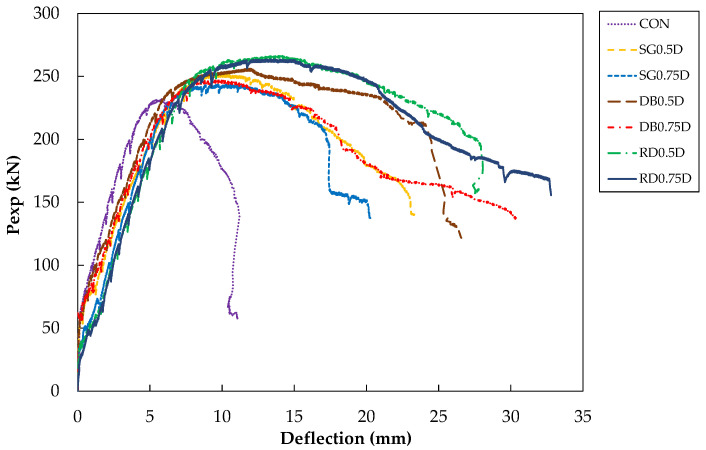
Load–deflection response of slab specimens (at slab center).

**Figure 9:**
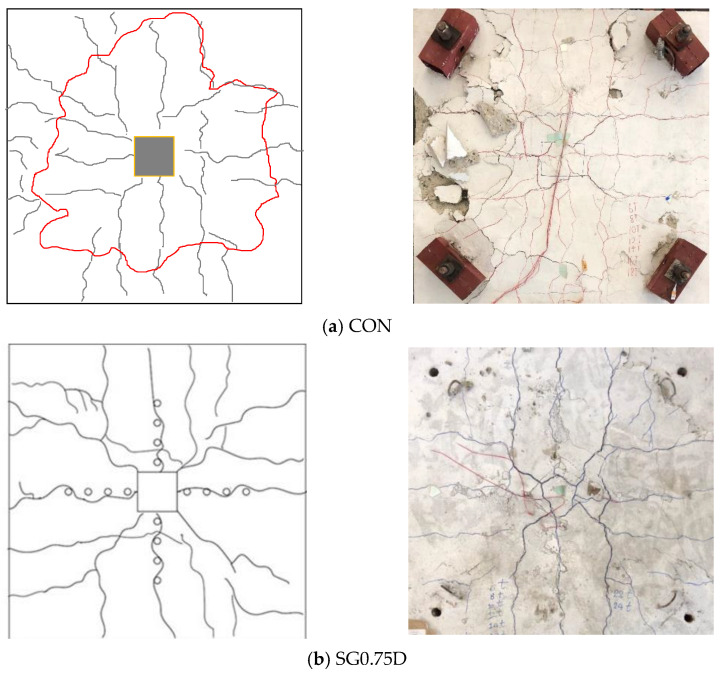

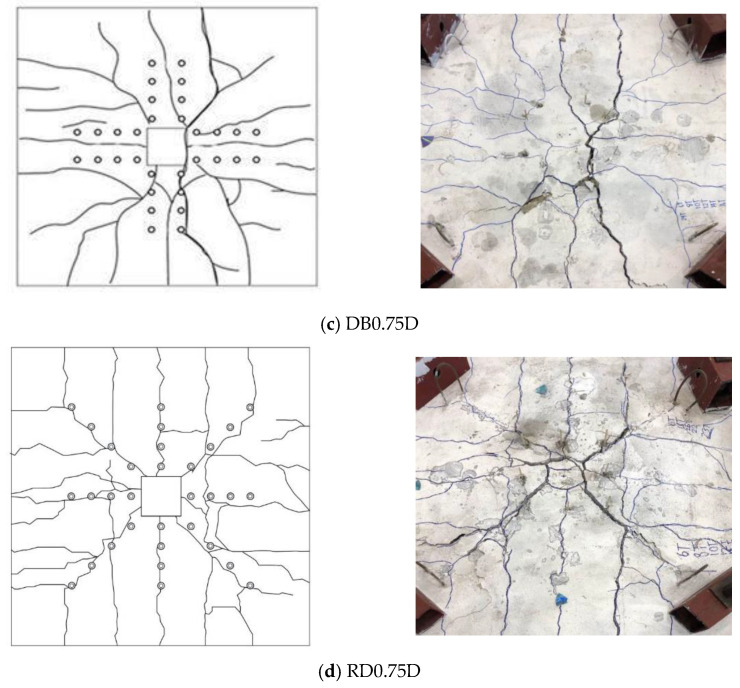
Schematic and observed crack patterns of test specimens (**a**) CON; (**b**) SG0.75D; (**c**) DB0.75D and (**d**) RD0.75D.

Experimental load–deflection curves at the center of the slabs are shown in Figure 8. Two types of failures were observed: punching shear and flexure failure. Control specimen was expected to fail in shear. On contemporary, strengthened specimens were expected to surpass punching shear capacity and exhibit flexure failures. Peak loads observed for each specimen are presented in Table 3. Control specimen reached a maximum load of 230 kN at a deflection of around 6 mm followed by an abrupt decline of capacity. This behavior is indicative of shear failure which is brittle and accompanies no signs before failure. Figure 9a shows observed crack patterns of the control specimen. The onset of the first crack occurred around the column perimeter at approximately 80 kN. Further increase in load accompanied radial cracks originating at the column’s corners and remained stable till 70% of the ultimate load. Beyond this point, circumferential cracks formed as highlighted by the red line in Figure 9a. These cracks moved towards the edges as the load increased. Finally, the control specimen failed in punching shear failure.

**Figure 10:**
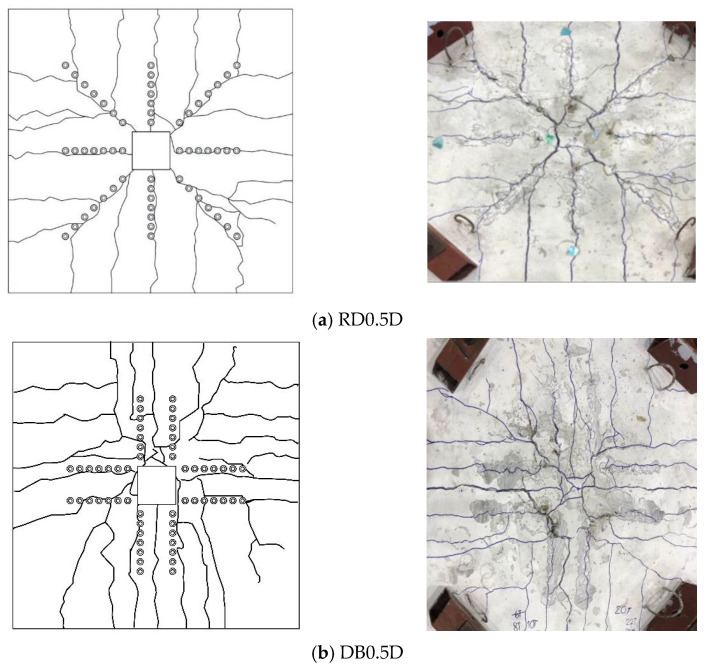

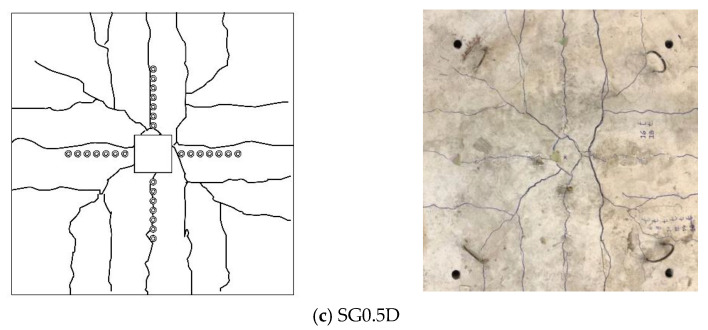
Schematic and observed crack patterns (**a**) RD0.5D; (**b**) DB0.5D and (**c**) SG0.5D.

Crack patterns of specimen SG0.75D are shown in Figure 9b. Load of 80 kN witnessed the onset of first cracks midway between the column’s sides near the GFRP drilled holes. Cracks migrated towards slab sides and were stable till 180 kN load. Further increase in load had a detrimental impact on cracks as they kept widening until the specimen reached its ultimate load at 243 kN. A significant improvement in the post-peak response of SG0.75D was observed in comparison to the control specimen. The ultimate load was increased up to 8.7% while ultimate deflection increased up to 81% for SG0.75D. Specimen DB0.75D exhibited similar crack patterns to those of SG0.75D (Figure 9c). However, cracks appeared only on three sides of the column and initiated between the two lines of GFRP rods. Migration of cracks towards slab edges was observed with a load increasing beyond 100 kN. Ultimate load and deflection were 247 kN and 30 mm, respectively corresponding to an improvement of 7.4% and 171% in ultimate load and deflection, respectively. Specimen RD0.75D incorporated the GFRP rods near the column’s corners and midway between the sides. Consequently, the onset of cracks was observed at both these locations that slowly migrated towards the slab’s edges as the load increased (see Figure 9d). Ultimate load and deflection were 264 kN and 33 mm, respectively, yielding improvements of 14.8% and 200% in the same order. Specimens in series 0.5D exhibited similar crack patterns to their counterparts in series 0.75D (Figure 10). The ultimate load of specimens SG0.5D, DB0.5D and RD0.5D were 251, 256 and 266 kN, respectively, yielding improvements of 9.1, 11.3 and 15.7%, respectively. Similar improvements in ultimate deflection for SG0.5D, DB0.5D and RD0.5D were 109, 136 and 154%, respectively.

Comparing the responses in two series, reducing the spacing of the GFRP rods had a benign effect on the ultimate load. Reducing the spacing from 0.75D to 0.5D resulted in an increase in ultimate load of 3.3, 3.6 and 0.8% for SG, DB and RD patterns, respectively. However, this did not ensure a similar pattern in terms of ultimate displacements.

### 3.2. Ductility

The term “ductility factor” is referred to in this study as the ratio of displacement at peak load $\delta_{u}$ to the displacement at a yield of longitudinal reinforcement $\delta_{y}$. Load displacement and strain distribution of steel bars were utilized to establish the yield displacement. As demonstrated in Table 3, the ductility factor of the control specimen could not be calculated as the steel bars did not reach a yield value of 0.0023. This is consistent with the failure mode of the control specimen which was controlled by the punching shear. Ductility factors of series 0.5D were consistently higher than those in series 1. A maximum ductility factor of 2.88 was observed for the specimen DB0.5D while DB0.75D created the lowest bound. These high ductility factors indicate that failure mode was successfully changed from shear to flexure. This is evident from the load-displacement curves of strengthened specimens (see Figure 8) which show a rather gradual descend in their post-peak response in comparison to the control specimen.

**Table 3:** Summary of test results.

Specimen ID	Peak Load (*kN*)	$\delta_{y}\;\left(mm\right)$	$\delta_{u}\;\left(mm\right)$	Ductility (μ)	Failure Mode	Energy Dissipation (kN-mm)
CON	230	/	5.8	/	S	1993.1
SG0.5D	251	4.59	10.1	2.20	F	4610.3
DB0.5D	256	4.07	11.8	2.89	F	5659.2
RD0.5D	266	/	13.7	/	F	6032.5
SG0.75D	243	6.21	9.6	1.55	F	3860.8
DB0.75D	247	7.54	9.3	1.24	F	5730.2
RD0.75D	264	9.26	12.78	1.41	F	6771.6

S for shear and F for flexure.

### 3.3. Initial Stiffness

The control specimen showed the highest initial stiffness of all as evident in load–deflection curves. Strengthening with GFRP rods resulted in a drop in the initial stiffness of all specimens as shown in Figure 8. This may be ascribed to the relatively lower modulus of GFRP rods than conventional steel. Another reason may be ascribed to the drilling of holes inside the slab for fixing the GFRP rods followed by epoxy soaking. Strengthened specimens showed similar initial stiffness and no clear trend to discriminate was detected.

### 3.4. Strain

**Figure 11:**
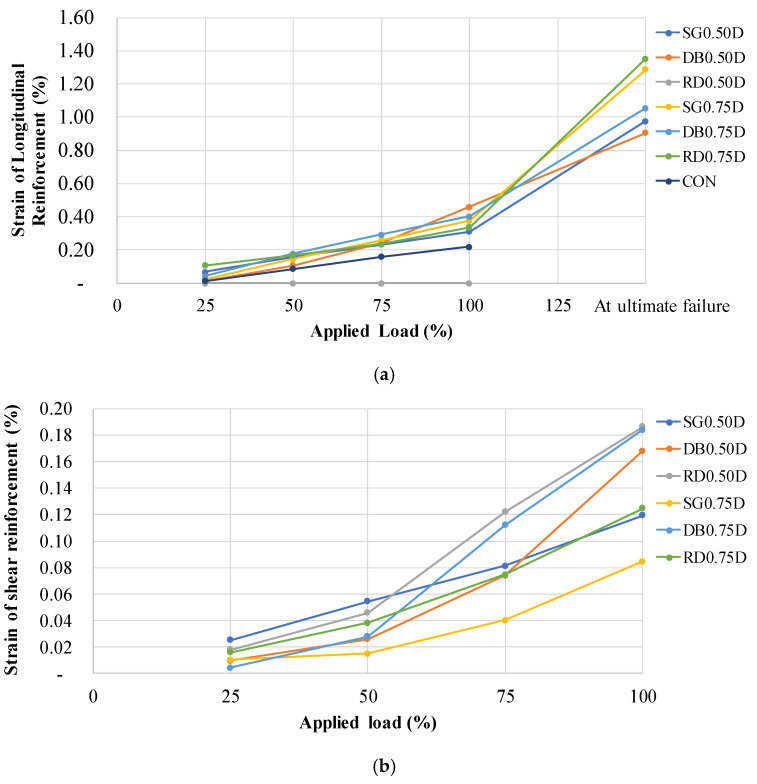
(**a**) Longitudinal steel strain vs. percentage peak load; (**b**) Shear steel strain vs. percentage peak load.

Longitudinal strain measurements corresponding to percentage peak load are presented in Table 4 and Figure 11a. The results show that the control specimen could not achieve the yield strain in longitudinal reinforcement. This is augmented by its load-deflection curve which is characterized by abrupt drop after peak load resulting in a brittle failure. It is to be mentioned that some malfunctioning of strain gauges inhibited strain measurements for specimen RD0.5D. Comparing strains in series 0.5D, specimen SG had regulated lower strain values than specimen DB at peak loads. This is substantiated by their corresponding ductility values as specimen DB0.5D exhibited 31.4% higher ductility than that of specimen SG0.5D. Nevertheless, post-peak response exhibited otherwise as specimen SG0.5D mobilized 7.7% higher longitudinal strains than specimen DB0.5D. The reason may be that a larger number of holes in the double pattern facilitated the propagation of cracks much quicker towards the slab edges. Series 0.75D showed a comparable pattern to those in series 0.5D. Specimen RD0.75D mobilized lowest strain values at peak followed by specimen SG0.75D and DB0.75D, respectively. Again, specimen DB0.75D mobilized the lowest post-peak strains. However, no such trend was shown by their corresponding ductility values. This supplements our earlier argument and that relatively larger spacings in series 0.75D did not facilitate crack propagation as much as it occurred in series 0.5D.

In terms of shear strain (Figure 11b), the lowest shear strains were mobilized in SG patterns in both series. Specimen RD0.5D mobilized higher strain values than specimen DB0.5D. However, the opposite was true in series 0.75D. This signifies that at reduced spacings, the radial pattern outperformed other patterns in terms of peak loads and ductility as GFRP rods were able to withstand higher strain values.

### 3.5. Energy Dissipation Capacity

High energy dissipation is a desirable property to ensure ductile mode of failure. The amount of energy dissipated by each specimen was evaluated by summing the area under load-deflection curves and corresponding values are tabulated in Table 3. As expected, the lowest energy dissipation was associated with the control specimen which showed a brittle post-peak response. In each series, the SG pattern formed the lowest bound of dissipated energy. Results indicate that reducing the spacings had a beneficial effect on energy dissipation for the SG pattern. Maximum energy was dissipated by specimen RD0.75D and contrary to SG specimens, reducing the spacing to 0.5D resulted in a 12.25% reduction of energy dissipation capacity. Analogous to this, the DB pattern also exhibited a 1.26% reduction in energy dissipation capacity as GFRP spacings reduced from 0.75D to 0.5D.

## 4. Analytical Validation

**Figure 12:**
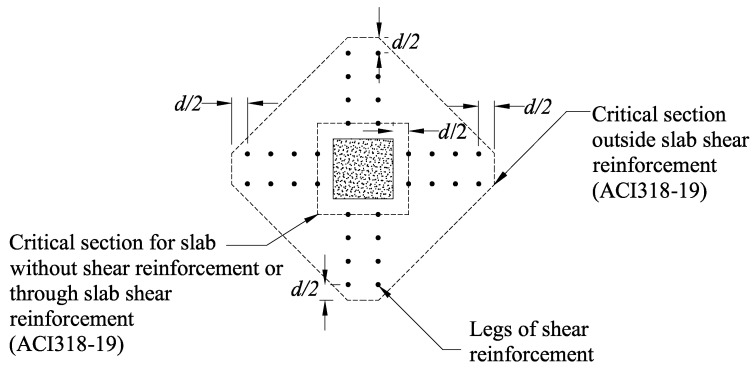
Definition of two-way shear critical sections in flat slabs.

ACI 318-14 (2014) proposed the use of a critical shear section for two-way shear in slabs with shear reinforcement. This critical section is located at an offset of 0.5d from the column’s face. Another critical section at an offset of 0.5d beyond the outermost peripheral line of shear reinforcement should also be considered. For instance, critical sections to be considered in the DB0.75D shear strengthening pattern are shown in Figure 12.

A shear-strengthened two-way slab can either fail within or outside the shear-strengthened zone depending upon the cumulative capacity of shear reinforcement and concrete.

### 4.1. Punching Shear Failure Inside the Shear-Strengthened Zone

ACI 318-14 proposes descriptive equations for shear capacity contributions from concrete $V_{c}$ and shear reinforcement $V_{s}$ as follows:

(1) $$\;V_{c}=min\left[\left(0.25\;\sqrt{{f^{\prime }}_{c}}\right),\;\left(0.083\left(\frac{\alpha_{s}d}{b_{0}}+2\right)\sqrt{{f^{\prime }}_{c}}\right),\;\left(0.167\left(1+\frac{2}{\beta_{c}}\right)\sqrt{{f^{\prime }}_{c}}\right)\right]$$

where:

- $f{’}_{c}$ = concrete cylinder compressive strength (MPa),
- d = effective slab thickness for shear (mm),
- $b_{o}$ = perimeter of shear critical section at 0.5d from loading area periphery (mm),
- $\alpha_{s}$ = Factor according to the type of connection; it is 40 for internal columns, 30 for external columns, and 20 for corner columns,
- $\beta_{c}$ = Ratio of the long side to the short side of the loading area periphery.

ACI 318-14 provides a descriptive equation for contribution in shear strength from steel studs as follows:

(2) $$\;V_{s}=\frac{A_{v}f_{yt}}{b_{0}s}$$

where:

- $A_{v}$ = sum of the area of all shear reinforcement in one peripheral line,
- $f_{yt}$ = Yield strength of shear reinforcement,
- s = spacing between consecutive peripheral lines of shear reinforcement parallel to loading area periphery.

Total shear strength is a summation of the contributions from concrete and steel with an upper limit as:

(3) $$V_{t}=V_{c}+V_{s}\leq 0.67\;\sqrt{{f^{\prime }}_{c}}\;MPa\;$$

Unlike steel, composite materials behave linearly in their stress–strain curves and do not possess any yield point. To incorporate the contribution of GFRP rods in total shear strength, Equation (2) is modified in this study. $A_{v}$ is replaced with $A_{GFRP}$ while $f_{yt}$ is replaced with $f_{u,GFRP}.$

Where:

- $A_{GFRP}$ = sum of the area of GFRP reinforcement in one peripheral line,
- $f_{u,GFRP}$ = fracture strength of the GFRP,

Results concluded that only 50% of GFRP strength was utilized in this study. Hence, the strength contribution from GFRP is given as:

(4) $$V_{GFRP}=\frac{0.5A_{GFRP}f_{u,GFRP}}{b_{o}s}$$

### 4.2. Punching Shear Failure Outside Shear-Strengthened Zone

ACI 318-14 (2014) proposes that the maximum shear strength is only provided by concrete ($V_{c}$) at a critical shear section offset from the outermost peripheral line of shear reinforcement equal to 0.5d. The corresponding equation is given as:

(5) $$\;V_{c}=\;0.167\sqrt{{f^{\prime }}_{c}}\;MPa\;$$

Table 5 and Table 6 presents detailed calculations of analytical calculations of shear strength as per above-mentioned approach and the comparison with experimental values.

As demonstrated in Table 6 (last column), a specimen with an SG pattern was unable to produce sufficient shear capacity to avoid punching shear failure within the shear-strengthened zone. On contrary, DB and RD patterns successfully shifted the punching shear failure outside their corresponding shear-strengthened zones. This is explained with their $V_{T}/V_{c,outside}$ ratios greater than 1.

## 5. Conclusions

This study aimed at strengthening flat slabs using post-installed GFRP rods. On this note, single, double, and radial at two spacings were investigated. The following conclusions may be drawn according to the results obtained from the experiments.

1. The load–deflection curve of the control specimen exhibited a sudden drop after attaining peak load. Strengthened specimens successfully shifted this failure mode to a gradual and ductile one. Improvements in both peak load and corresponding deflections were observed. Reducing the spacing from 0.75D to 0.5D had a beneficial impact on peak load for each GFRP pattern.
2. Strain measurement indicated no yielding of longitudinal bars in the control specimen. On the contrary, all strengthened specimens exhibited yielding. The highest strain values at peak loads were mobilized in double GFRP patterns followed by single and radial patterns, respectively. In terms of shear strain, the lowest shear strains were mobilized in SG patterns in both series. Specimen RD0.5D mobilized higher strain values than specimen DB0.5D. However, the opposite was true in series 0.75D. This implies that at reduced spacings, the radial pattern outperformed other patterns in terms of peak loads and ductility as the GFRP rods were able to withstand higher strain values.
3. As expected, the control specimen did not show any ductility. Nevertheless, strengthening with GFRP rods developed ductility. Reducing the GFRP spacing resulted in an increase in ductility irrespective of the type of GFRP pattern.
4. In each series, the SG pattern formed the lowest bound of dissipated energy. Results indicate that reducing the spacings had a beneficial effect on energy dissipation for the SG pattern. Maximum energy was dissipated by specimen RD0.75D and contrary to SG specimens, reducing the spacing to 0.5D resulted in a 12.25% reduction of energy dissipation capacity. Analogous to this, the DB pattern also exhibited a 1.26% reduction in energy dissipation capacity as GFRP spacings reduced from 0.75D to 0.5D.
5. It can be concluded that the use of GFRP rods is beneficial in the remediation of punching shear failure and GFRP rods can be effectively used in existing structures to improve the structural response of flat slabs.
6. ACI 318-14 equations for two-way shear strength were modified to incorporate shear strength contributions from the GFRP. A comparison of analytical results with experimental results suggests that the proposed approach is successful in predicting the shear strength capacity of flat slabs in the presence of GFRP rods.

Based on experimental results, GFRP bars can be effectively utilized in enhancing the shear strength of flat slabs. Mode of failure becomes ductile with increased peak loads, ductility and energy dissipation capacities as compared to control specimens. Of the three GFRP patterns studied, the radial pattern provided maximum improvements in terms of peak loads and energy dissipation capacities. This is the only pattern that was least affected by GFRP rods’ spacing.

## 6. Future Recommendations

This study did not take the effect of the diameter of GFRP rods into account. It is assumed that larger diameter GFRP rods can result in larger required spacing. Analogous to this, a thicker flat slab would require a lesser amount of GFRP rods. By taking all potential parameters into account, a complete model predicting the required amount of GFRP rods for a given flat slab system can be established.

## Figures and Tables

**Table 1:** Mechanical properties of reinforcements.

Reinforcement	Diameter (mm)	Elastic Modulus (GPa)	Yield Strength (MPa)	Tensile Strength (MPa)
Steel Bars	16	210	488	652
GFRP	6	35.6	/	500

**Table 2:** Details of test specimens.

Specimen ID	Slab Thickness (mm)	Effective Depth (mm)	Flexural Reinforcement	Shear Reinforcement			
				$\frac{s_{o}}{d}$	$\frac{s}{d}$	No.	Size (mm)
CON	150	114	7DB-16@200	/	/	/	/
SG0.5D	150	114	7DB-16@200	0.4	0.5	7	6
DB0.5D	150	114	7DB-16@200	0.4	0.5	7	6
RD0.5D	150	114	7DB-16@200	0.4	0.5	7	6
SG0.75D	150	114	7DB-16@200	0.4	0.75	4	6
DB0.75D	150	114	7DB-16@200	0.4	0.75	4	6
RD0.75D	150	114	7DB-16@200	0.4	0.75	4	6

$s_{o}=\mathrm{Spacing}\;\mathrm{between}\;\mathrm{column}’s\;\mathrm{face}\;\mathrm{and}\;\mathrm{first}\;\mathrm{shear}\;\mathrm{reinforcement}$. s=Spacing between shear reinforcement. d=slab’s effective depth.

**Table 4:** Strain readings at various percentages of peak loads.

Specimen ID	Percentage Strain at Percentage Peak Load								Failure
	25		50		75		100		
	L	S	L	S	L	S	L	S	
CON	0.01	/	0.09	/	0.16	/	0.22	/	/
SG0.50D	0.07	0.03	0.16	0.05	0.23	0.08	0.31	0.12	0.98
DB0.50D	0.01	0.01	0.11	0.03	0.24	0.07	0.46	0.17	0.91
RD0.50D	/	0.02	/	0.05	/	0.12	/	0.19	/
SG0.75D	0.02	0.01	0.14	0.02	0.26	0.04	0.37	0.08	1.29
DB0.75D	0.04	0.0	0.18	0.03	0.29	0.11	0.40	0.18	1.06
RD0.75D	0.11	0.02	0.17	0.04	0.23	0.07	0.34	0.12	1.35

S = Shear Strain. L = Longitudinal Strain.

**Table 5:** Comparison of theoretical ($V_{T})$ and experimental total shear strength.

Specimen ID	$b_{o}\;\left(mm\right)$	$A_{GFRP}\;\left(mm^{2}\right)$	$V_{c}\;\left(kN\right)\;\mathrm{Equation}\;(1)$	$V_{GFRP}\;\left(kN\right)\;\mathrm{Equation}\;(4)$	$V_{T}\;\left(kN\right)\;\mathrm{Equation}\;(3)$	$\frac{V_{T}}{P_{exp}}$
CON	1256	/	229.15	/	229.15	1.00
SG0.50D	1256	113.10	173.60	56.47	230.07	0.92
DB0.50D	1256	226.19	173.60	112.94	286.54	1.12
RD0.50D	1256	226.19	173.60	112.94	286.54	1.08
SG0.75D	1256	113.10	173.60	37.43	211.03	0.87
DB0.75D	1256	226.19	173.60	74.86	248.46	1.01
RD0.75D	1256	226.19	173.60	74.86	248.46	0.94

$A_{GFRP}$ = sum of the area of GFRP reinforcement in one peripheral line. $V_{GFRP}$ = shear strength contribution from *GFRP*. $V_{c}$ = shear strength contribution from concrete. $b_{o}$ = critical perimeter. $V_{T}$ = theoretical shear strength. $P_{exp}$ = experimental shear strength.

**Table 6:** Prediction of failure zones using analytical results.

Specimen ID	$b_{outside}\;$ (mm)	$V_{c,outside}\;$ (kN)	$\frac{V_{T}}{V_{c,outside}}$
CON	/	/	/
SG0.50D	2916	269.23	0.85
DB0.50D	2971	274.31	1.04
RD0.50D	3048	281.42	1.02
SG0.75D	2560	236.36	0.89
DB0.75D	2610	240.98	1.03
RD0.75D	2680	247.44	1.00

$b_{outside}\;$ = critical perimeter outside shear-strengthened zone. $V_{c,outside}\;$= shear strength contribution from concrete (Equation (5)).$\;V_{T}$ = total shear strength within shear-strengthened zone.

## Data Availability

The data presented in this study are available on request from the corresponding author.
